# Double Burdens or Double Resilience: A Qualitative Study of Chinese Older Couples both with Multimorbidity

**DOI:** 10.1093/geroni/igaf122.131

**Published:** 2025-12-31

**Authors:** Yuanyuan Jin, Yu Zhang

**Affiliations:** Soochow University, Suzhou, Jiangsu, China (People’s Republic); Soochow University, Suzhou, Jiangsu, China (People’s Republic)

## Abstract

As the population ages, the occurrence of older adults and their spouses both living with multimorbidity is becoming increasingly common. Multimorbidity not only affects the patients themselves but also imposes a heavy burden on family caregivers. When both partners experience multimorbidity, caregiving and care-receiving roles often blur. Limited research exists on the lived experiences and daily interactions of such couples. This study aimed to explore the lived experiences and daily interactions of older couples both with multimorbidity. A qualitative study was conducted in China from May 2023 to January 2025, involving 20 couples. Reflexive thematic analysis was used to analyze data. Four key themes were generated: triadic relationship, dynamic daily health management interactions, double burdens, and double resilience. In older couples where both partners have multimorbidity, roles of “patient” and “caregiver” shift based on acute illness flares, declining function, or third-party involvement. Daily health management interactions reflect independence, interdependence, or dependence, shaped by health status and preferences. Double burdens include physical tolls, financial hardship, and emotional contagion. Conversely, double resilience is reflected in the formation of a nudge effect, the generation of emotional resonance, and the encouragement of family-wide advocacy for a healthy lifestyle. This study adopts a dyadic perspective to explore the experiences and interactions within older couples where both partners experience multimorbidity. Formal or informal caregiving support from third parties, as well as the nudge effect and emotional resonance between spouses, play a pivotal role in offering essential support, helping older couples better navigate the challenges of multimorbidity.

